# Accuracy of surface strain measurements from transmission electron microscopy images of nanoparticles

**DOI:** 10.1186/s40679-017-0047-0

**Published:** 2017-10-25

**Authors:** Jacob Madsen, Pei Liu, Jakob B. Wagner, Thomas W. Hansen, Jakob Schiøtz

**Affiliations:** 1 Department of Physics, Technical University of Denmark, Fysikvej, Building 311, 2800 Kongens Lyngby, Denmark; 2 Center for Electron Nanoscopy, Technical University of Denmark, Fysikvej, Building 311, 2800 Kongens Lyngby, Denmark

**Keywords:** High-resolution transmission electron microscopy, Strain mapping, Nanoparticles, Surface strain

## Abstract

Strain analysis from high-resolution transmission electron microscopy (HRTEM) images offers a convenient tool for measuring strain in materials at the atomic scale. In this paper we present a theoretical study of the precision and accuracy of surface strain measurements directly from aberration-corrected HRTEM images. We examine the influence of defocus, crystal tilt and noise, and find that absolute errors of at least 1–2% strain should be expected. The model structures include surface relaxations determined using molecular dynamics, and we show that this is important for correctly evaluating the errors introduced by image aberrations.

**Electronic supplementary material:**

The online version of this article (doi:10.1186/s40679-017-0047-0) contains supplementary material, which is available to authorized users.

## Background

The surface lattice strain in nanostructures as a topic of research has gained increased interest in recent years due to its significant impact on many material properties. As an example, surface strain is a possible tunable parameter that can be used to optimize the adsorption energies of surfaces for a particular catalytic reaction [1]. Platinum-based oxygen reduction catalysis is improved by weakening the binding of adsorbed oxygen intermediates by 0.1 eV, this can be achieved by a 2% compressive strain [2]. Strain in nanoparticles can be generated by a variety of sources: particle size, shape, twinning, by the lattice mismatch between metals in multimetallic core–shell nanoparticles or it can be induced by the supporting substrate [3]. Characterizing the influence of these effects requires a technique capable of measuring structural information at atomic resolution.

High-resolution transmission electron microscopy (HRTEM) has become a routine tool for determining the structure of materials at an atomic scale [4]. TEM is particularly attractive due to the ability to map local strain. However, TEM images are the result of a complex diffraction and aberration-limited imaging process, and hence considerable care needs to be shown when extracting quantitative information.

An approach to overcome this is to iteratively compare experimental images with simulations [5, 6]; imaging parameters and model structure of the sample are refined until the simulated and experimental image match. This method has been successfully applied to determine various structures including surfaces. Another solution is to reconstruct the exit wave from a focal series, to eliminate the effect of aberrations [7]. However, the additional complexity added by such methods has limited their use. Instead an often used approach is to obtain the atomic positions directly from the experimental images. The positions of the intensity extrema within the image depend on imaging conditions, orientation and sample thickness, hence they do not necessarily coincide with the atomic positions. However, in the periodic part of a solid, a constant spatial relationship can still be assumed between the image and the atomic positions. This assumption breaks in areas with thickness variations, defects and in particular in the vicinity of surfaces and interfaces [8] and thus a systematic assessment of the accuracy is needed for these cases.

A first investigation to determine the accuracy with which surface strain could be determined was undertaken by Marks [9]. Image simulations were used to compare actual relaxations, in the input structural models, with apparent relaxations, measured from the corresponding simulated images. He found that there was a linear relationship between apparent and real strain, with a constant outward shift of about 5%. He also demonstrated that the true positions of atomic columns at the surface could be determined within 0.2 Å, corresponding to 5% of the lattice parameter of gold. This investigation was done before the invention of the spherical aberration corrector, which today has made it feasible to measure surface relaxations on the order of a few percent.

Newer investigations on the accuracy of strain analysis directly from HRTEM images have focused on interfaces in heterostructures [8, 10–12]. The error in such cases was found to be as low as 0.5% [13, 14]. Using a new technique based on Fourier transforming several overlapping sliding windows, it has been demonstrated that picometric precision and accuracy of interatomic distances can be achieved for measurements inside periodic solids [15]. However, these studies do not investigate surfaces and generally assume a uniform thickness. Moreover, in all these cases the strain distributions were fundamentally 2D, i.e. the atomic columns were mainly displaced in the plane perpendicular to the zone axis. This is different from nanoparticles where the true 3D strain is projected as a 2D image.

The literature has several examples of studies using aberration-corrected microscopy that includes measurements of strain in nanoparticles, and in the vicinity of surfaces, these measurements are often backed by comparison with a simulation that approximates the experimental structure and microscope conditions [16–19]. The general conclusion is that the erroneous surface strain due to imaging aberrations is much smaller in aberration-corrected images than the 5% found by Marks. However, these studies lack a systematic analysis of the sensitivity to experimental variables.

In the present work, we evaluate the accuracy of strain analysis directly from simulations of aberration-corrected HRTEM images focusing on surfaces of nanoparticles. The simulated objects are gold nanoparticles, which in addition to being a topic of research in their own right, provides a model structure that has different exposed surfaces and a linear thickness gradient. We examine the influence of four different effects: defocus, particle size, crystal tilt and noise, and we investigate what accuracy can be expected under which imaging and sample conditions.

## Methods

### Image simulation

#### Model and temperature effects

**Fig. 1:**
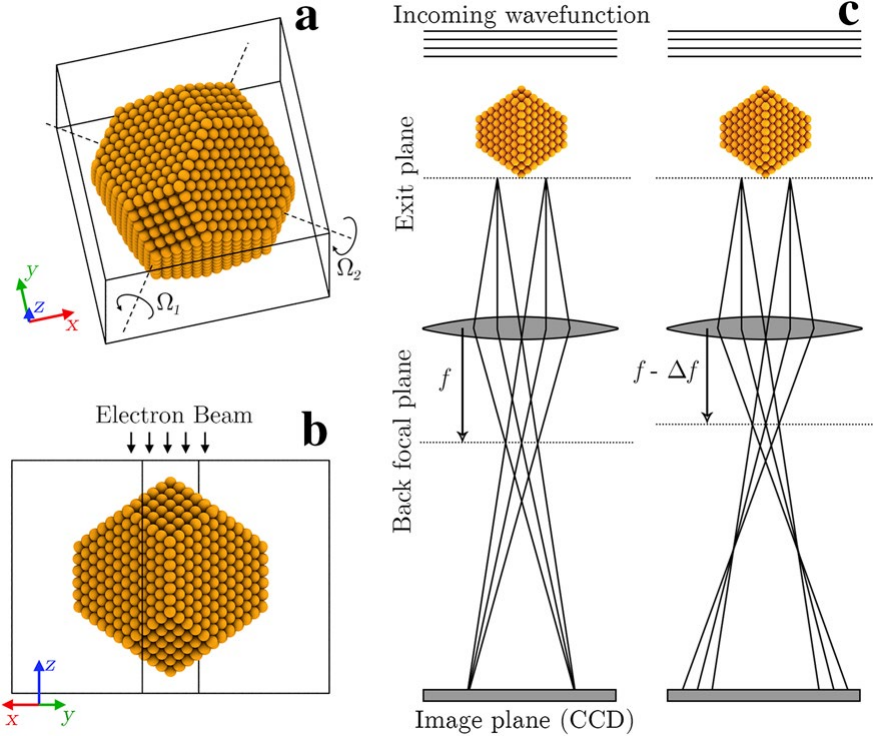
**a**, **b** Model gold nanoparticle containing 1925 atoms with a diameter of  4 nm. The electron beam travels in the negative *z*-direction. The full lines indicate the computational cell and the dashed lines indicate the rotation axes denoted $\Omega _1$ and $\Omega _2$. **c** The HRTEM images are simulated by propagating the incoming plane wave through the sample potential using the multislice algorithm. The resulting wave at the exit plane is transferred through the objective lens to the detector using the CTF. Defocus is given relative to the bottom of the nanoparticle, with a positive defocus referring to propagation toward the detector

The overall shape of the model clusters was determined using Wulff constructions. The models were placed in a computational cell with 5 Å vacuum on all sides of the particle, see Fig. 1. Real metal surfaces are not simply ideally truncated crystals; experimental studies have demonstrated that the surface layer of many clean transition metals relaxes inward [20], while expansion of the top layer has been found for some surfaces of noble metals [21], including the {111} facets of gold. It has been proposed that expansive surface strains in small decahedral gold nanoparticles are a contributor to their catalytic activity [22].

In this study the ideal crystals were relaxed using molecular dynamics (MD) with an empirical potential. The interactions between the atoms were calculated with the charge-optimized many body (COMB) potential [23]. The potential parameters were fitted with a high priority for surfaces and nanoparticles, and hence reproduce the experimental surface relaxations of gold quite well. For an infinitely extended {111} surface, the potential predicts a 1.2% surface expansion of the top layer, which is close to the experimental value of 1.3% [24]. For {100} surfaces an inward relaxation of 1.1% is predicted. There is no corresponding experimental value; however, the prediction is close to 1.2% [25] and 1.51% [26] calculated with density functional theory.

The effect of finite temperature is included using the frozen phonon approximation [27]. This is a semi-classical model based on the assumption that a single high-energy electron passing through the specimen at about half the speed of light can only probe a single frozen “snapshot” of the vibrating crystal. The image is produced by averaging incoherently over many snapshots where the atoms are slightly displaced from their equilibrium positions. The frozen phonon model has been shown to be numerically equivalent to the full quantum-mechanical treatment of the inelastic phonon scattering process [28]. The snapshots are typically determined using the Einstein approximation; however, we chose to use random steps from a constant temperature MD simulation using Langevin dynamics at 300 K [29]. We only used steps after the initial equilibration and the simulation was run for long enough to properly represent the thermal distribution of the atomic positions. We found that the simulated images are converged when $\sim 40$ snapshots are included in the averaging.

During a MD simulation the projected atomic positions follow a 2D normal distribution. The standard deviation of this distribution is around 0.05 Å or approximately 2% of the distance between the columns. The standard deviation of the distributions is not identical for all columns. It can be approximately 30–40% larger for some surface and corner atoms (see Additional file 1: Figure S3). We find that the difference between the mean relaxed positions and the mean positions obtained from a thermal average is just a constant thermal expansion of the entire crystal.

#### Diffraction and objective lens aberrations

The exit waves were simulated with the multislice algorithm [30] using the QSTEM code [31]. This code has been interfaced with Python and utilizes the atomic simulation environment [32] for setting up model structures, providing a single environment for building models, simulating and analysing images. The code is publicly available.^1^ We have also made code available for directly recalculating and analysing a selection of the results from this paper.

The electrostatic potential of the sample was generated using the independent atom model with the parametrizations of Rez et al. [33]. The potential was generated on a 3D grid before slicing, allowing for accurate simulations of tilted samples. Aberrations due to the objective lens were included by Fourier space multiplication with the contrast transfer function (CTF). The effect of a finite source size and energy spread (i.e. partial spatial and temporal coherence) was included in the Quasi-coherent approximation where envelopes are applied to the wave function [34]. The imaging process is illustrated in Fig. 1c.

The microscope conditions were modelled after an image aberration-corrected FEI Titan microscope operated at 300 kV. Unless otherwise stated, the third-order spherical aberrations were set to $C_{\text{s}} = -10$ $\upmu$m and all other aberrations except for defocus were set to zero. Other aberrations are generally not negligible in aberration-corrected microscopy; however, we chose to neglect them in order to keep the degrees of freedom limited. We tested the stability of our results to inclusion of additional aberrations, in particular twofold astigmatism on the order of 5–10 nm and 5th-order spherical aberrations on the order of 2.5 mm. While some results change slightly, we found that inclusion of additional aberrations does not change our conclusions in significant ways.

The focal spread was $\Delta = 2.9$ nm and the convergence angle was set to 15 mrad. The sampling used for the simulations was at least 0.05 Å/pixel, and when needed the large simulated images were downsampled using bilinear interpolation.

#### MTF and thermal magnetic noise

A single electron can cause a signal in more than one pixel of the CCD due to multiple scattering in the scintillator material. This effect can be described by the modulation-transfer function (MTF). A typical MTF can be parametrized as the sum of a Gaussian and an exponential [35]

1 $$\begin{aligned}{\text{ MTF}}(q) = a \exp (-b q) + (1 - a)\exp (-c^2 q^2) , \end{aligned}$$

where *q* is the spatial frequency and the parameters are taken as $a=0.58$, $b=2.5$ ${\AA }$ and $c=5.9$ ${\AA }$.

An additional blurring can be caused by all kinds of noise that lead to a random deflection of the image relative to the detector. The origin of these aberrations are vibrations and drift of the stage, time-dependent fields resulting from instabilities of the lens currents and in particular thermal magnetic noise resulting from magnetic fields due to eddy currents in the material of the lenses [36]. The blurring is modelled by a Gaussian envelope on the intensity distribution [37]

2 $$\begin{aligned} \mathcal {N}(q) = \exp (-(2\pi \sigma )^2 q^2 ) , \end{aligned}$$

where $\sigma$ denotes the standard deviation, and a value of $\sigma = 0.25$ Å has been assumed. It has been shown that including the MTF and a Gaussian blur can account for the so-called Stobbs factor [38], the ubiquitous contrast mismatch between experimental and simulated images [39]. Since these effects can drastically reduce the contrast, they are important to include for accurately quantifying the influence of noise.

#### Finite electron dose

We assume that the noise is dominated by shot noise, and hence the measured electron count in each pixel can be modelled by a Poisson distribution [40]. The average number of electrons *N* collected by the *i*th detector pixel is given by

3 $$\begin{aligned} N_i = D \delta ^2 I_i , \end{aligned}$$

where *D* is the dose in electrons per area,* δ* is the sampling and * I*_*i*_ is the probability for an electron hitting the *i*’th pixel. The signal-to-noise ratio of the whole image is given by [41]

4 $${\text{SNR}} = \frac{{\bar{N}}}{{\sigma (N)}},$$

where ${\bar{N}}$ is the average number of electrons per pixel and $\sigma (N)$ is the standard deviation of the number of electrons collected by each pixel. In the limit of low dose this can be reduced to [42]

5 $${\text{SNR}} = \sqrt {\bar{N}} = \sqrt {DI} \delta,$$

whereas in the limit of high dose other sources of noise are dominant (e.g. thermal noise) and the SNR becomes constant. We are only including shot noise in the simulations.

### Strain analysis

There are several different approaches for obtaining strain directly from HRTEM images. The methods can broadly be classified into three different types: direct measurement of interatomic distances in real space [43, 44], extraction of the lattice by comparison to a template [45] and analysis in Fourier space [46]. The results of the different approaches are similar inside periodic structures, but can differ in the presence of defects [44]. In this paper the real space method is used, since it has the most straight forward interpretation for surfaces, where the results of Fourier space analysis are very opaque. A comparison between real and Fourier space analysis, using geometric phase analysis (GPA), is provided as supplementary information (see Additional file 1: Figure S4).

The most critical step in the real space approach is to determine the positions of the lattice points. There are several ways of defining these positions. However, the simplest way is to define them as the position of the intensity extrema, assumed to correspond with an atomic column. If the lattice points do not correspond to single intensity peaks, they can instead be found using a cross-correlation of the image with a template motif [8]. The intensity extrema are found at sub-pixel accuracy by fitting a 2D function, usually a polynomial or a Gaussian, to the neighbourhood of each peak and setting the derivatives to zero [44]. It is also possible to define the lattice positions from the centre of mass of the intensity distributions [47]. The methods agree if the intensity distributions are symmetric. However, this is not necessarily the case close to asymmetries in the lattice, such as an interface. A comparison of the two methods of measuring the atomic positions is included as supplementary information. The conclusion is that the methods lead to slightly different errors; however, the magnitude of the errors is essentially the same.

The peak pairs algorithm [44] is the most popular method for finding strain from a set of 2D lattice points from HRTEM images. For the calculation of strain at every lattice point, the peak pairs algorithm uses only two lattice vectors. We have found that an approach using a larger number of lattice vectors is significantly more stable in the presence of noise. For an fcc crystal in the [48] zone axis, this method uses the four nearest and two second nearest neighbours to find the strain at any lattice point in the bulk. Another advantage of this method is that it allows us to determine the strain for lattice points at all surfaces and corners, which is not possible with the standard implementation of the peak pairs algorithm. The routines used for strain analysis, including a rudimentary implementation of GPA, are implemented in Python and made available as open source.^2^

**Fig. 2:**
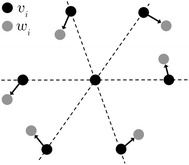
The black points indicate the ideal lattice for an fcc crystal in the [48] zone axis. The grey points are the positions of slightly displaced lattice points for a strained crystal. The strain at the central lattice point is calculated by finding the optimal affine transformation between the black and grey points, denoted by *v* and *w*, respectively

The strain is computed at each lattice point, by comparing the positions of the neighbouring lattice points in an ideal template lattice to the corresponding measured lattice points. In practice, this is done by finding the optimal affine transformation, ${\mathbf{A}}$, between the two sets of vectors, see Fig. 2. In general finding ${\mathbf{A}}$ is an overdetermined problem, hence it is found as the best fit to a least-squares fit of the form:

6 $$\begin{aligned} r= {\mathrm{min}}_{\mathbf{A}} \sum _i^N \left\| {\mathbf{A}}{\textbf{v}}_i-{\textbf {w}}_i \right\|, \end{aligned}$$

where *r* is the residual term, $v_i$ and $w_i$ are vectors containing the ideal and actual lattice vectors, ${\mathbf{A}}$ is the affine transformation and $\Vert \cdot \Vert$ denotes the Euclidean norm. The orientation and elastic strain matrices can be extracted from ${\mathbf{A}}$ via a left-sided polar decomposition of the deformation gradient

7 $$\begin{aligned} \textbf {P}\textbf {U}={\mathbf{A}}, \end{aligned}$$

where ${\textbf{U}}$ is an orthogonal right-handed matrix (the rotation matrix), and ${\textbf{P}}$ is a symmetric matrix (the elastic strain matrix). Finding the correspondence between ${\varvec{v}}$ and ${\varvec{w}}$ is done using a branch and bound search method. A similar 3D equivalent of the method is described by Larsen et al. [48].

To limit the amount of results that have to be shown, we will usually just show the planar strain, $\epsilon _{\text{p}}$, calculated as the average of the normal strains in the *x*- and *y*-direction

8 $$\begin{aligned} \epsilon _{\text{p}} = \frac{1}{2}(\epsilon _{xx} + \epsilon _{yy}) . \end{aligned}$$

Surface relaxations are the strain at the outermost atoms in the direction perpendicular to the same surface. Hence, the surface relaxation associated with an atom on a surface perpendicular to the unit vector $\hat{\varvec{n}}$ is found as

9 $$\begin{aligned} \epsilon _{\hat{\varvec{n}}} = \hat{\varvec{n}}^{\text{T}}\varvec{\epsilon }\hat{\varvec{n}} . \end{aligned}$$

We are mainly interested in the strain measurement errors, but to define the errors, we first need to define the true strain. An image provides a single viewpoint of the structure, where each atomic column appears as a dot, hence we can only hope to measure an average column position for the atoms belonging to each column. Defining these averages to be the true column positions, the corresponding planar strain will be denoted as $\epsilon _{{\text{p}},\,{\text{true}}}$. The strain calculated from the positions of the maxima in the matching image will be denoted as$\epsilon _{{\text{p}},\,{\text{measured}}}$. From these definitions, we define the error of a strain measurement as

10 $$\begin{aligned} \mathrm {error}\,(\epsilon _{\text{p}}) = \epsilon _{{\text{p}},\,{\text{measured}}}-\epsilon _{{\text{p}},\,{\text{true}}} . \end{aligned}$$

## Results

### Influence of relaxations and temperature effects

When image simulations are used to estimate errors due to aberrations, it is a common practice to use a model of an unrelaxed crystal, under the assumption that the errors caused by these aberrations are insensitive to the small difference between the unrelaxed and relaxed crystal [16–19]. Our results demonstrate that this assumption is invalid in general.

**Fig. 3:**
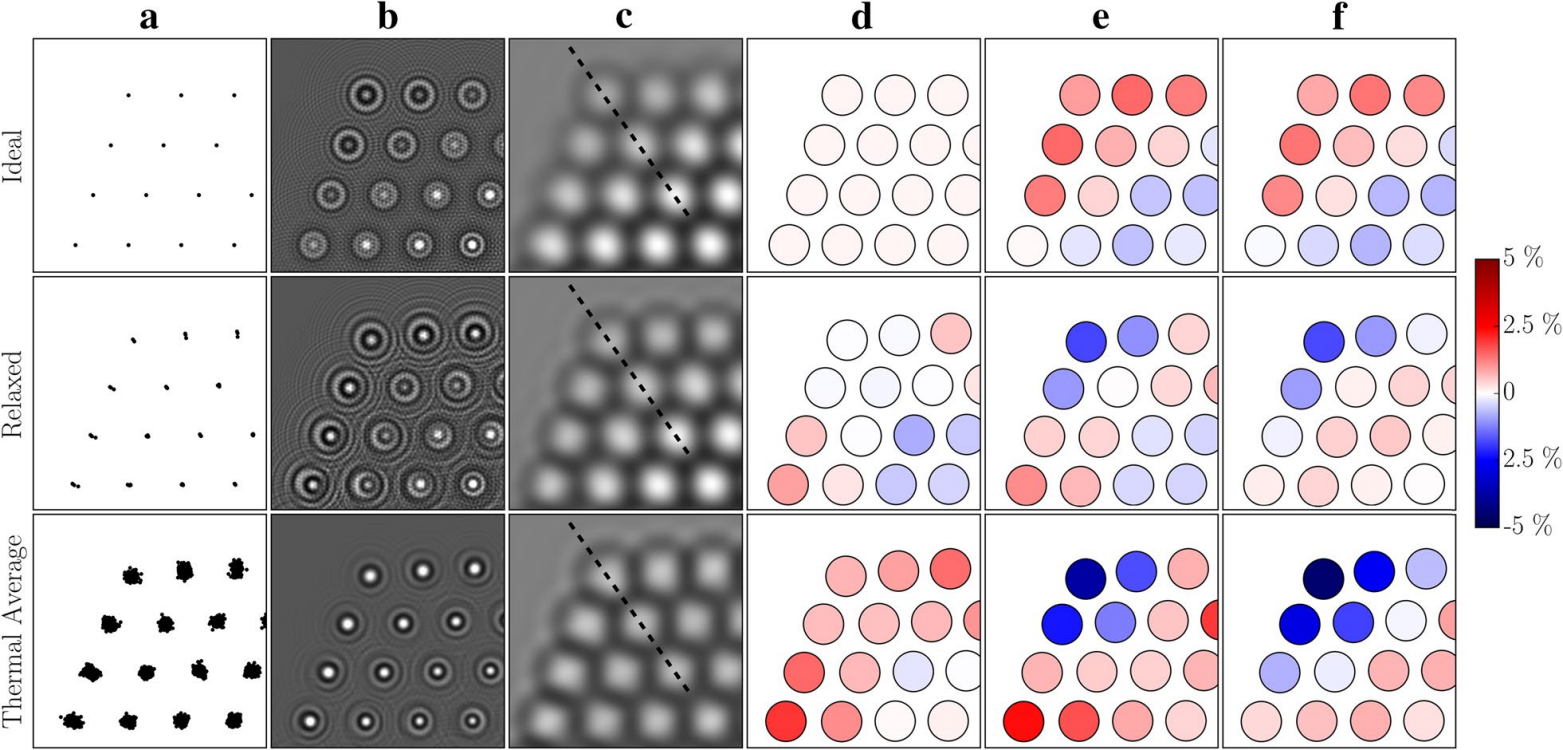
From top to bottom, the rows contain results relating to an ideal crystal, a relaxed crystal and a thermal average of crystals. Each of the panels show a small section of the corner between two {111} facets. Along the columns we show: **a** the projected positions of the atoms, all the positions used in the thermal average are included. **b** Intensities of the exit waves. **c** Simulated images for a defocus $\Delta f = 14.5$ nm. **d** The true planar strain, $\epsilon _{\text{p},\,{\text{true}}}$, i.e. the strain calculated directly from the projected positions of the model crystal. The colour coding shared by all the columns is shown to the right of the figure. **e** The measured planar strain, $\epsilon _{{\text{p}},\,{\text{measured}}}$, i.e. strain calculated from the measured positions of the intensity maxima in simulated images. **f** The measurement error of the planar strain, ${\mathrm {error}}(\epsilon _p)$, calculated as the difference between the strain shown in the two preceding columns

The comparison in Fig. 3 shows the difference between results based on an ideal crystal, a relaxed crystal and an average over thermal vibrations. There is a substantial difference between the exit wave intensities. This difference is less obvious in the final images; however, it is large enough to have an impact on the measured strain and more notably on the measurement errors. This means that using the ideal particle to calibrate a strain measurement would lead to wrong conclusions about the measurement errors.

**Fig. 4:**
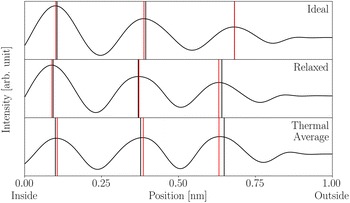
Slices along the dashed lines in Fig. 3. The black vertical lines indicate the true atomic positions and the red vertical lines indicate the corresponding measured maxima positions. For the ideal model the measured distance between the outermost peaks is too large by 0.006 nm or 2% of the interatomic distance in the slice direction. For the images that include temperature effects, the same measurement is too small by 0.025 nm or 10% of the interatomic distance

The origin of the errors is deviations from the constant spatial relationship between the image and the underlying projected potential. The peaks are generally more asymmetric for both the image resulting from a relaxed crystal and from a thermal average of crystals, and these small irregularities in the symmetry of adjacent intensity peaks can cause large measurement errors, as illustrated in Fig. 4. All results in the following sections will be based on simulations where temperature effects are included. We also note the $\sim 50$% reduction of the image contrast due to thermal vibrations, making the influence of temperature on the image contrast approximately as important as the MTF and thermal magnetic noise.

**Fig. 5:**
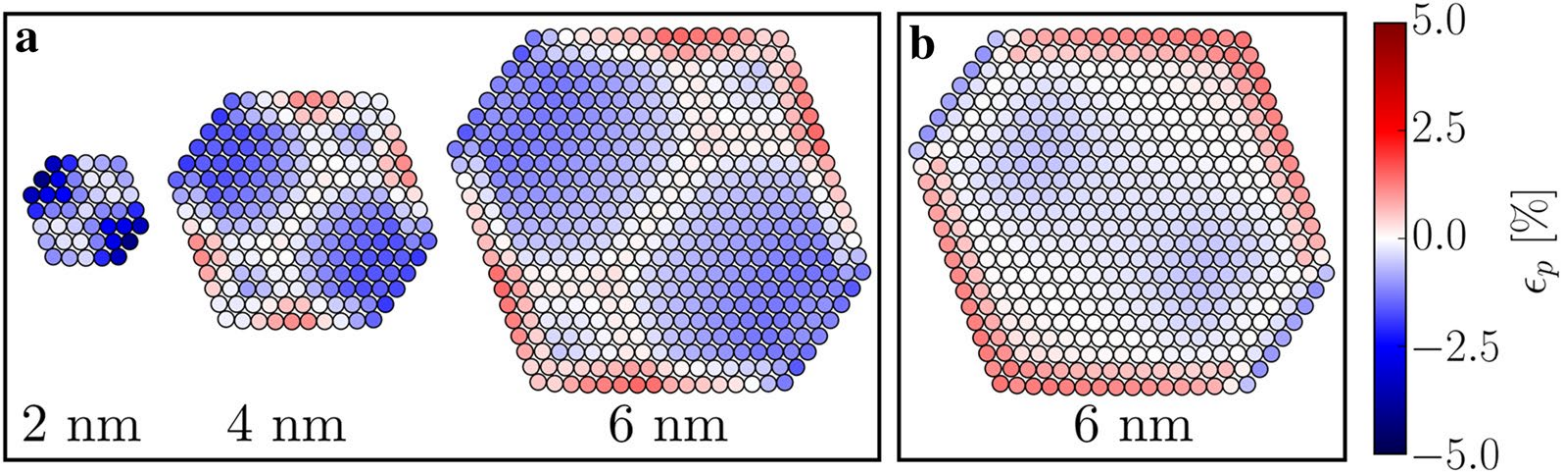
**a** The “true” planar strain, $\epsilon _{\text{p},\,{\text{true}}}$, calculated from the average projected column positions of the model, for three different nanoparticle diameters. **b** The actual planar strain for a slice through the 3D model

The strain calculated from the true average projected column positions is shown in Fig. 5a for three different particle diameters. The strain calculated from the projected positions seems to show a significant compressive strain in the bulk of the particle; however, this is misleading. Figure 5b shows the strain calculated directly from the full 3D model for a slice through the centre of the nanoparticle; comparing the strain in the 3D model to the projected strain reveals that the apparent bulk compressive strain is due to relaxations closer to the front and back surface. Hence, even disregarding image aberrations, comparing Fig. 5a, b shows that care has to be taken, when interpreting strain measurements from HRTEM images. The errors in the following sections are calculated with respect to the strain in the projected positions and are thus mainly due to image aberrations.

### Influence of defocus

**Fig. 6:**
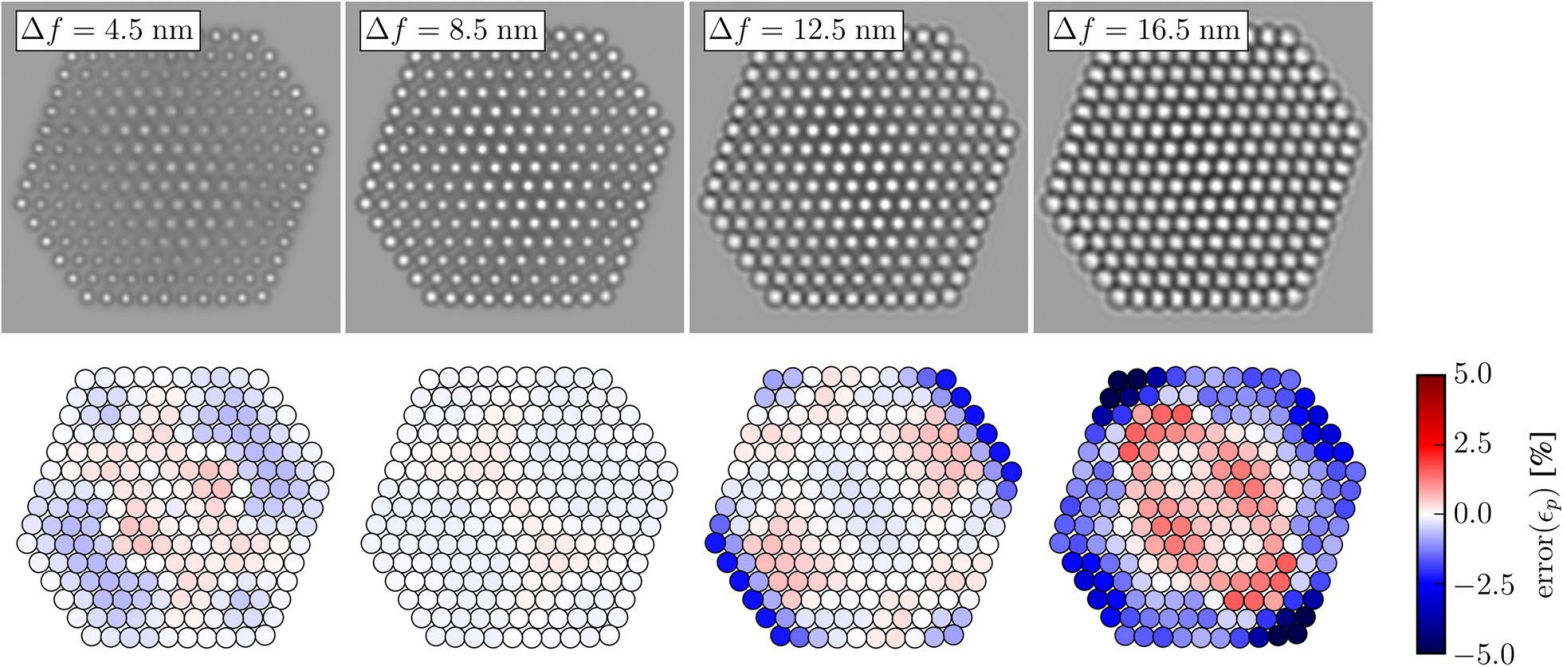
The top row shows simulated images for a nanoparticle with a diameter of 4 nm. The bottom row shows the corresponding distribution of errors in the planar strain. The defocus is different in each column, as indicated in the figure

The top row of Fig. 6 shows simulated images at different defocus and the bottom row shows the error in the planar strain measured from these images. The smallest defocus shown is 4.5 nm since contrast inversion begins to take effect for a smaller defocus. We present results for only a positive defocus, which leads to images with bright spots at the positions of the atomic columns. We have obtained results for negative defocus as well, where the atoms appear as black spots on a lighter background. The results are shown in Additional file 1: Figure S5.

A defocus of 8.5 nm results in planar strain errors smaller than 1% everywhere, while a defocus of 12.5 nm causes significant errors at the {100} facets. Due to the sign and location of these errors, they could easily be mistaken for real surface relaxations. The errors generally stay small for columns not at the surface; however, at larger defocus some errors start to appear, generally following the thickness gradient.

**Fig. 7:**
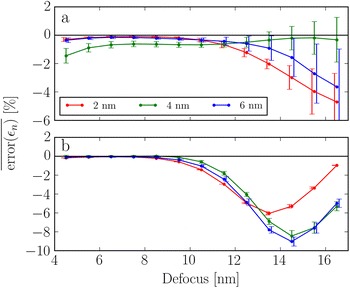
The error in the measured surface relaxations averaged across the facets as a function of defocus, for the three particle sizes given in the legend, for: **a** the {111} facets and **b** the {100} facets. The bars indicate the standard deviation of the errors in the surface relaxation error across the facets. The bars are shifted slightly from the points for visual clarity

The error in the measured surface relaxations averaged across the facets for the uppermost atomic layers is shown as a function of defocus in Fig. 7. Since this error can vary quite a bit across the {111} facets, we also show the corresponding standard deviation. Results for 3 different particle sizes are shown, from a diameter of $\sim 2$ nm to a diameter of $\sim 6$ nm.

For the {100} facet the error is almost zero up to a defocus of 8.5 nm, across all three particle sizes. Meanwhile the error for the {111} facet never becomes smaller than 1% for the 4 nm particle, which is approximately the same magnitude as the actual relaxations. For both facets and all sizes, the errors stay below 2% up to a defocus of $\sim 11$ nm, where the mean error increases sharply at the {100} facets. The mean error does not increase as drastically for the {111} facets. On the other hand, the standard deviation does increase. This is mainly due to the thickness variation along these facets.

### Influence of tilt

**Fig. 8:**
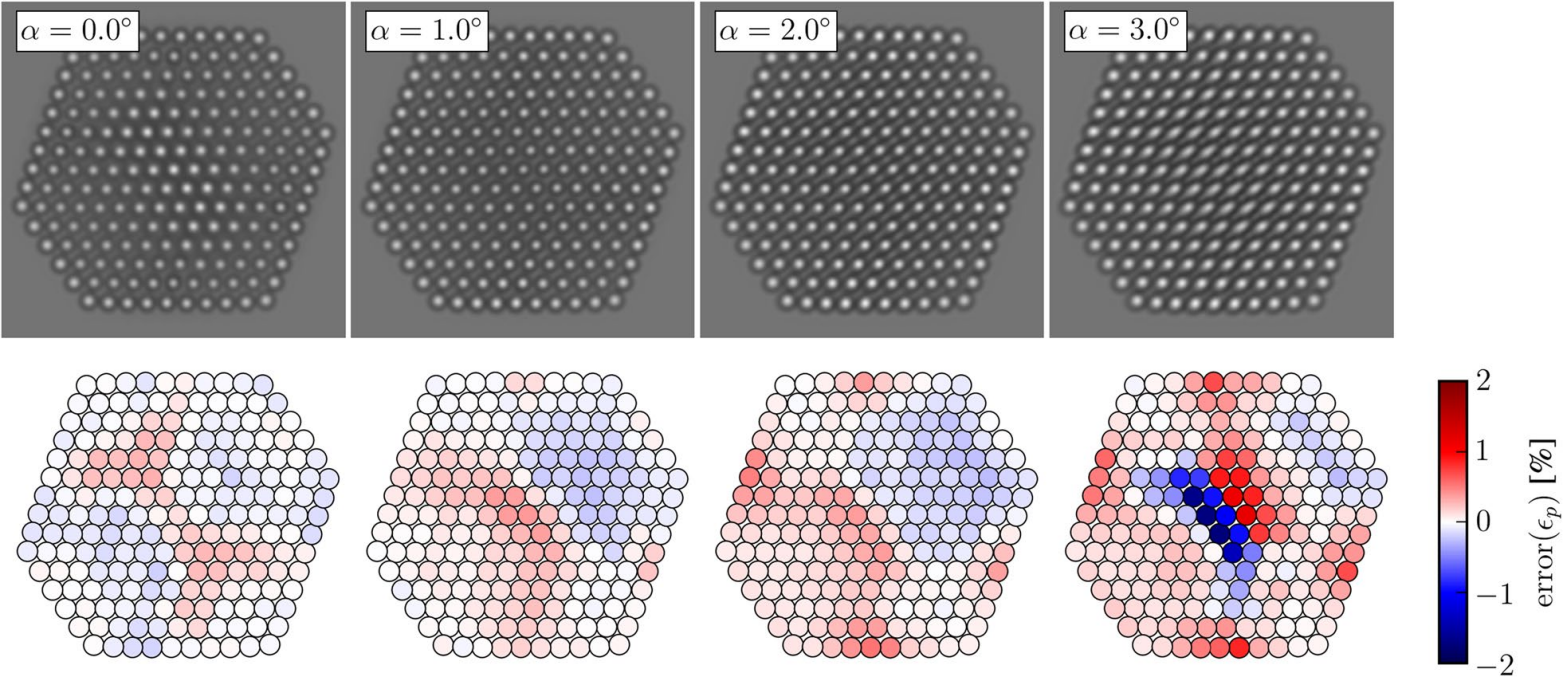
The top row shows simulated images at increasing tilt around the $\Omega _1$-axis for a defocus $\Delta f = 8.5$ nm. The bottom row shows the error in the planar strain at each lattice point measured from these images

**Fig. 9:**
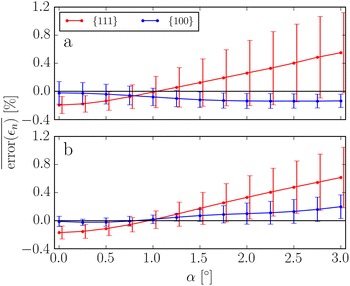
The error in the measured surface relaxations averaged across the facets as a function of tilt, around the axes (**a**) $\Omega _1$ and (**b**) $\Omega _2$. The defocus was $\Delta f = 8.5$ nm. The curves are for the {100} and {111} facets, as indicated by the legend

It is unavoidable that the sample will be slightly tilted relative to the ideal zone axis. Figure 8 shows the distribution of errors in the planar strain for increasing tilt, $\alpha$, around the $\Omega _1$-axis. At tilt $\alpha =1.0^{\circ }$, the errors have changed very little compared to the untilted crystal, though the appearance of the image have changed in the central part of particle, this is due to an effective diminishing of the projected potential, as have been reported elsewhere [49]. The errors stay small up to a tilt $\alpha =2.0^{\circ }$, but increase sharply in the centre of the nanoparticle between $\alpha =2.0^{\circ }$ and $\alpha =3.0^{\circ }$. The error introduced by tilt is very dependent on the height of the atomic columns, since the length of the footprint of the projection of a tilted column increases linearly with its height. Only one direction of tilt is shown; however, the trends are similar for other tilt directions. One other tilt directions is included as Additional file 1: Figure S6.

Figure 9 shows the effects of tilt on the errors in the measured surface relaxations for a defocus $\Delta f = 8.5$ nm. The tilt has a relatively limited impact on the measured surface relaxations. The mean and standard deviation of error changes by at most 1% over the entire tilt range. The effects of tilt on the strain measurements are very dependent on defocus. For example at a defocus $\Delta f = 14.5$ nm, the mean surface relaxation error changes by more than 6% at the {100} facets, a plot showing this is shown in Additional file 1: Figure S7.

### Influence of noise

**Fig. 10:**
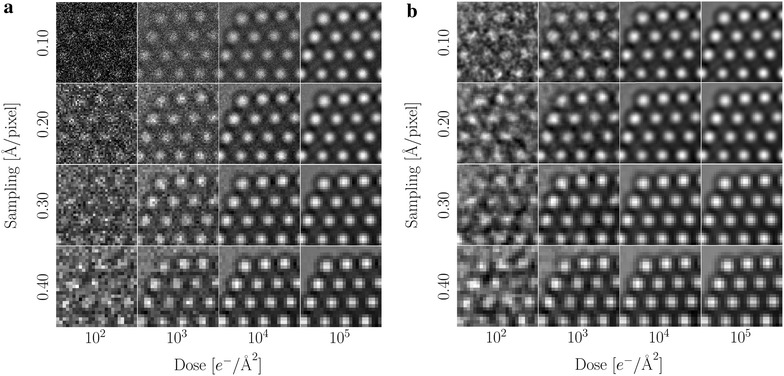
**a** Sections of the simulated images at a defocus of $\Delta f = 8.5$ nm for different doses and samplings. All images are mapped onto the same range of grey levels. **b** The same images after applying a Wiener filter

The evolution of the object visibility with respect to the sampling and dose is shown in Fig. 10a. At a dose of $10^2$ $e^-/{\AA}^2$ the object is barely visible, while the images are essentially unaffected by noise at $10^5$ $e^-/{\AA}^2$.

Noise removal is essential to obtain the stable polynomial fits necessary for sub-pixel resolution; hence we show the same noisy images after application of a Wiener filter in Fig. 10b [50]. The regularization of the filter was chosen to be optimal for each of the different samplings, but was not changed with the amount of noise.

**Fig. 11:**
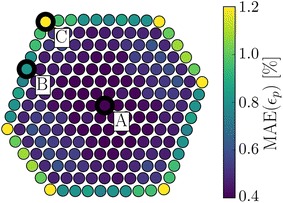
The MAE of the planar strain due to noise at each lattice point for a Wiener filtered noisy image at a sampling of 0.2 Å/pixel, a dose of $10^3$ $e^{-}/{\AA }^2$ and a defocus $\Delta f = 8.5$ nm

To determine the influence of dose on the errors in strain measurements, we simulate a statistically representative ensemble of images, $K=300$, with different distributions of noise. The error due to noise is quantified using the mean absolute error, MAE, over the ensemble of images for each lattice point

11 $$\begin{aligned} \mathrm {MAE}_{i} = \frac{1}{K}\sum _{k=0}^K \left| \epsilon _{k,i} - \epsilon _{\infty ,i} \right| , \end{aligned}$$

where $\epsilon _{k,i}$ is the planar strain at the *i*’th lattice point measured from the *k*th noisy image and $\epsilon _{\infty ,i}$ is the corresponding measured strain without noise. Since the automatic polynomial fitting can fail at low doses, extreme outliers have been removed before taking the average. Figure 11 shows the distribution of the MAE across a nanoparticle, there is a fairly large difference between the MAE for different lattice points, varying by a factor of three between the centre of the particle and a corner. The reason for this is mainly that the strain at surfaces is determined on the basis of fewer surrounding lattice points. The strain at corner atoms is determined on the basis of just three neighbours, while the measurements in the centre rely on twice that number of neighbours.

**Fig. 12:**
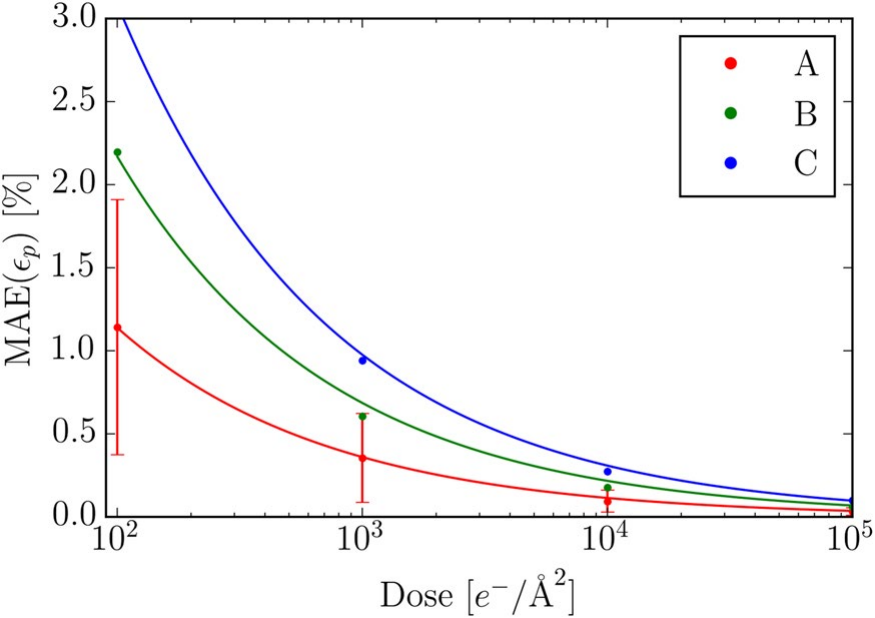
The MAE as a function of the dose for the three lattice points, *A*, *B* and *C*, as indicated in Fig. 11. The dots show the MAE calculated from the simulated images and the full lines are curves of the form given by Eq. (12), where the constant of proportionality has been fitted to the dots. The bars indicate the standard deviations, which for visual clarity are shown only for lattice point *B*, proportionally the standard deviations are similar for the other lattice points

The MAE at three chosen lattice sites as a function of dose is shown in Fig. 12. We find a simple approximate empirical relationship, assuming constant sampling, between the MAE and the dose

12 $$\begin{aligned} \mathrm {MAE} \propto \frac{1}{\sqrt{D}} \propto \frac{1}{\mathrm {SNR}} , \end{aligned}$$

where the constant of proportionality is determined by the number of neighbours, local image contrast and sampling. The second approximate proportionality assumes low dose and is due to Eq. (5).

**Fig. 13:**
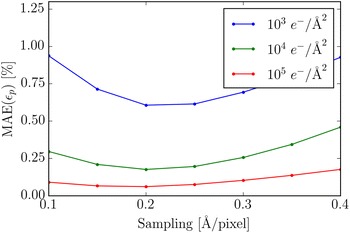
The MAE as a function of the sampling for five different doses at the *B* lattice point (see Fig. 11). The defocus was $\Delta f = 8.5$ nm and the sampling was 0.2 ${\AA }/$pixel

Given that the SNR depends linearly on the sampling [see Eq. (5)], the expression above might lead one to expect that coarser sampling would give smaller MAE. This is however not the case, as shown in Fig. 13 where the MAE is plotted as a function of sampling for different doses. The relationship is fairly constant though a sampling of 0.2 Å/pixel is better than both a rougher or a finer sampling. The main reason that there is no decrease in the MAE as the sampling gets coarser is that the better SNR is compensated by a smaller number of pixels across each peak available for polynomial fitting.

**Fig. 14:**
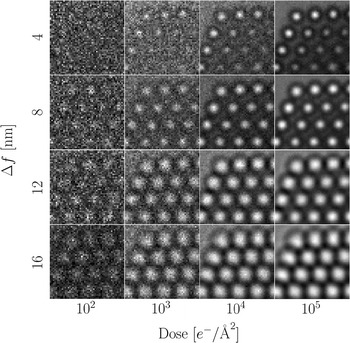
Sections of simulated HRTEM images for different doses and defocus at a sampling of 0.2 ${\AA}$/pixel. All images are mapped onto the same range of greys

**Fig. 15:**
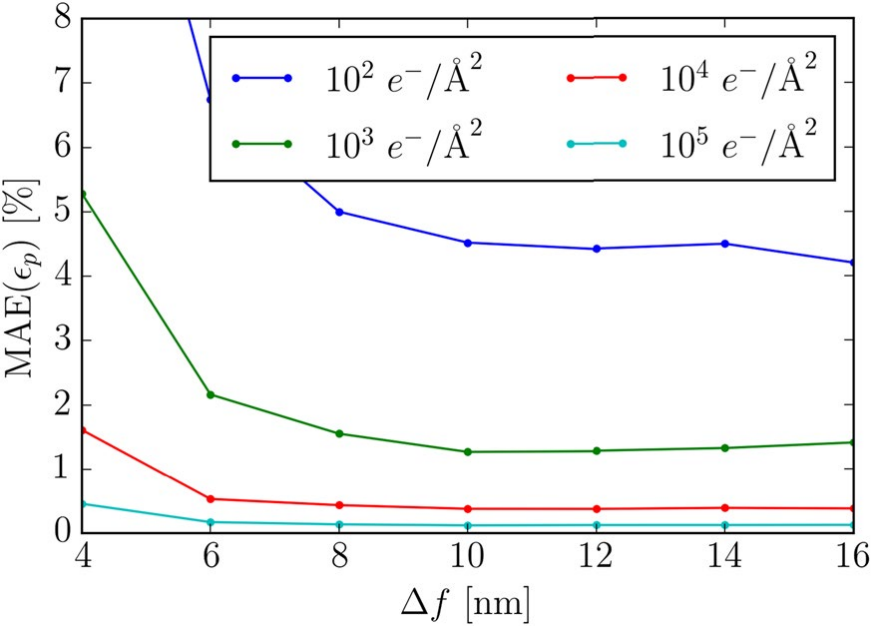
The MAE as a function of defocus for four different doses at a sampling of 0.2 ${\AA}$/pixel at the lattice point B

In the previous sections, we saw that the defocus should be kept small to obtain strain measurements that are relatively unaffected by aberrations. The disadvantage of this is that phase contrast imaging relies on the additional phase added by the objective lens, and hence a too small defocus will negatively impact the image contrast. This effect is illustrated in Fig. 14 where the change in the visibility of the nanoparticle is shown with respect to defocus and dose. The corresponding errors are quantified in Fig. 15. At a low dose, the errors grow very large when the defocus is small, but even at a higher dose, errors due to noise become present when the defocus is too small. When the defocus is increased the MAE becomes smaller, however saturation is reached relatively quickly, and additional defocus beyond $\Delta f=8$ nm does not further improve the MAE.

## Conclusion

We looked at the accuracy of surface strain measurements from HRTEM images of nanoparticles. We showed that the practice of using simulations based on ideal sample models to calibrate strain measurements is problematic, since the predicted errors from such simulations do not in general reflect the errors for an identical model that includes relaxations.

In general, the impact of the interaction between tilt, thickness and defocus on the final strain measurement is very complicated. However, we observe that if the defocus is small enough, the errors in the measured surface relaxations due to image aberrations can be kept at less than 2%, even for visually obvious tilts. This is significantly larger than the 0.5% that have been found for strain measurements inside periodic solids [13]. The main reason for the larger error is the asymmetry in the peaks close to surfaces.

In order to obtain measurements with small errors, the defocus should not be chosen solely to maximize contrast, since this will also cause large errors due to aberrations. The choice of defocus has to balance delocalization and contrast; if the defocus is too small the contrast will suffer, while if defocus is too large the image aberrations will be the main source of error.

For a dose of $10^3$ $e^-/{\AA}^2$, the optimal defocus for the gold nanoparticles is somewhere around 8.5 nm; at this defocus the errors in the surface relaxations are below 2% and the expected noise error is 1.2% with a standard deviation 0.8%.

## Additional file

**Additional file 1.** **Section S1.** Measuring the center of mass. **Figure S1.** Definition of integration regions for center of mass calculations. **Figure S2.** Comparison of center of mass positions with peak positions. **Figure S3.** Magnitudes of thermal vibrations. **Figure S4.** Comparison of our method with GPA. **Figure S5.** Negative defocus measurements. **Figure S6.** Planar strain errors for increasing tilt. **Figure S7.** Surface strain errors for increasing tilt.

## Footnotes

1
https://github.com/jacobjma/PyQSTEM.

2
https://github.com/jacobjma/structural-template-mapping.
