# Correction to: Accuracy of surface strain measurements from transmission electron microscopy images of nanoparticles

**DOI:** 10.1186/s40679-017-0049-y

**Published:** 2017-11-07

**Authors:** Jacob Madsen, Pei Liu, Jakob B. Wagner, Thomas W. Hansen, Jakob Schiøtz

**Affiliations:** 1 Department of Physics, Technical University of Denmark, Fysikvej, Building 311, 2800 Kongens Lyngby, Denmark; 2 Center for Electron Nanoscopy, Technical University of Denmark, Fysikvej, Building 311, 2800 Kongens Lyngby, Denmark

## Correction to: Adv Struct Chem Imag (2017) 3:14 10.1186/s40679-017-0047-0

Unfortunately, after publication of this article [1], it was noticed that the name of the fifth author was incorrectly displayed as Jakob Schiøz. The correct name is Jakob Schiøtz and can be seen in the corrected author list above. The original article has also been updated to correct this error.
