# Supplementary material for: Accuracy of surface strain measurements from transmission electron microscopy images of nanoparticles
Source: Adv Struct Chem Imaging. 2017 Oct 25;3(1):14. doi: 10.1186/s40679-017-0047-0 (PMC5656738; doi:10.1186/s40679-017-0047-0)
Supplement: Supplementary file 1 — Additional file 1. Section S1. Measuring the center of mass. Figure S1. Definition of integration regions for center of mass calculations. Figure S2. Comparison of center of mass positions with peak positions. Figure S3. Magnitudes of thermal vibrations. Figure S4. Comparison of our method with GPA. Figure S5. Negative defocus measurements. Figure S6. Planar strain errors for increasing tilt. Figure S7. Surface strain errors for increasing tilt. [file 40679_2017_47_MOESM1_ESM.pdf]

Supplementary Information: Accuracy of Surface Strain Measurements from Transmission Electron Microscopy Images of Nanoparticles

Jacob Madsen*[1], Pei Liu[2], Jakob B Wagner[2], Thomas W Hansen[2], and Jakob Schiøtz[1]

[1]Department of Physics, Technical University of Denmark
[2]Center for Electron Nanoscopy, Technical University of Denmark

August 7, 2017

S1 Center of mass

There are two natural ways of measuring the position of an atomic column. Either as the position of the intensity extrema or as the center of mass of the peak. We chose to use the extrema in the main text, as this is the simplest to understand and introduce less ambiguity compared to the center of mass.

Calculating the center of mass introduces the issue of choosing the integration region. One choice is to use a fixed integration region around each peak, however this method is very sensitive to how the fixed regions are chosen. We find that this introduces too much ambiguity. Another choice is to bound integration region by the contour lines of the image corresponding to some threshold value, e.g. the image mean. However, a problem arises when the size of the intensity valleys and peaks varies over the image. This can be solved by subtracting a local mean, I_B, which we choose to be the result of blurring the image with a Gaussian function

$$I_B(\boldsymbol{r}) = I(\boldsymbol{r}) * \left(\frac{1}{2\pi\sigma^2} \exp\left(-\frac{r^2}{2\sigma^2} \right) \right) , \tag{1}$$

where $I(\boldsymbol{r})$ is the image intensity as a function of the spatial coordinate $\boldsymbol{r} = (x, y)$, $*$ denotes a convolution and σ is the standard deviation of the Gaussian. Hence, the center of mass of the i'th peak is found as

$$\boldsymbol{C}_i = \frac{1}{A} \iint_{S_i} (I(\boldsymbol{r}) - I_B(\boldsymbol{r}))\boldsymbol{r} d\boldsymbol{r} , \tag{2}$$

where S_i is the region enclosing the peak bounded by $I - I_B = 0$, see Fig. S1(b). The method have one free parameter, σ, however the result is not very sensitive to small changes of this parameter, assuming it is larger than the inter column distances.

There is no theoretical basis for choosing the center of mass over the peak or vice versa, and generally the methods perform similarly. The maxima is very sensitive to small irregularities in the shape of the peak, however such irregularities can also change the integration region which in turn has an influence on the center of mass. The discrepancy between the two methods are typically on the same order as the error due to aberrations, see Fig. S2.

*Correspondence: jamad@fysik.dtu.dk

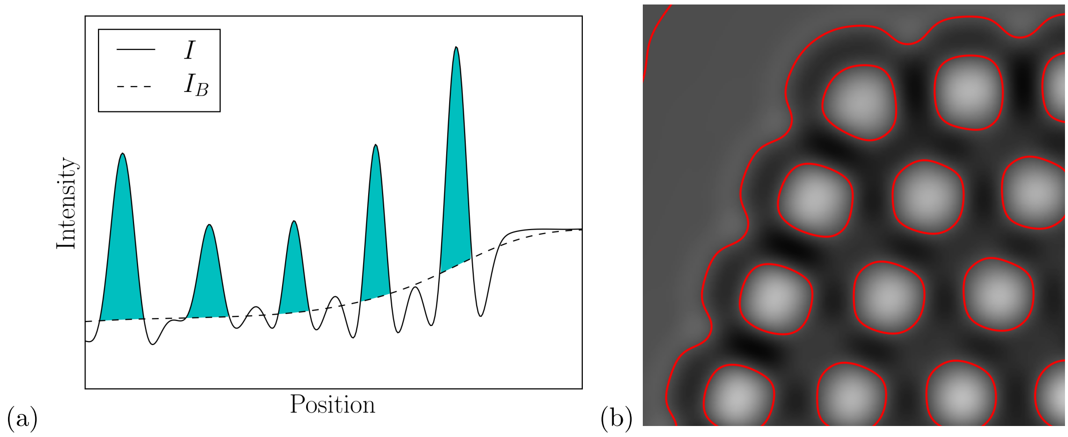

Figure S1: (a) The center of mass of each peak is found by integrating over the blue regions formed as the difference between the image intensity I and the local mean I_B. (b) The image intensity with the local mean subtracted, $I - I_B$, with the red lines indicating the contour corresponding to $I - I_B = 0$.

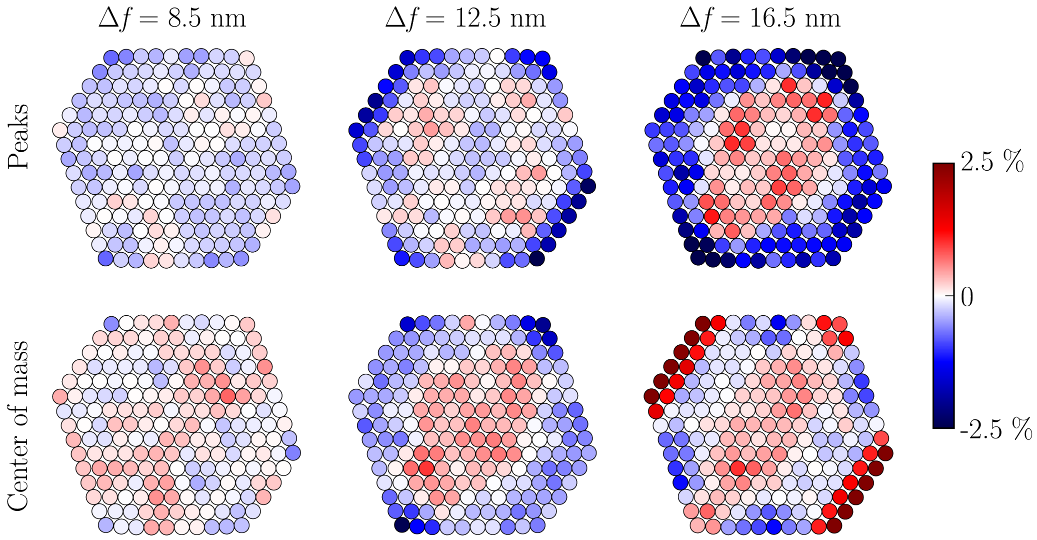

Figure S2: The error in the planar strain as measured using the peak maxima and center of mass. The input images correspond to those of Fig. 6 in the main text, with the exception that the electron dose was 5×10^3 $e^-/\text{Å}^2$.

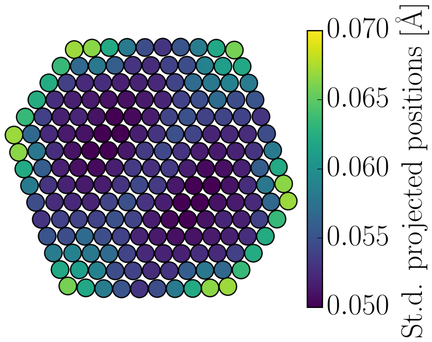

Figure S3: The thermal vibrations are predicted using molecular dynamics, hence the vibrational amplitude of the atoms can vary across the structure. Here we show the standard deviation of the distributions of projected column positions for a thermal ensemble of 40 nanoparticle structures from a constant temperature MD simulation at 300 K. A standard deviation of 0.05 Å is equivalent to approximately 2 % of the intercolumn distance in the [110] zone axis. The standard deviation is larger for surface and corner columns, reflecting the larger vibrational amplitude for these atoms.

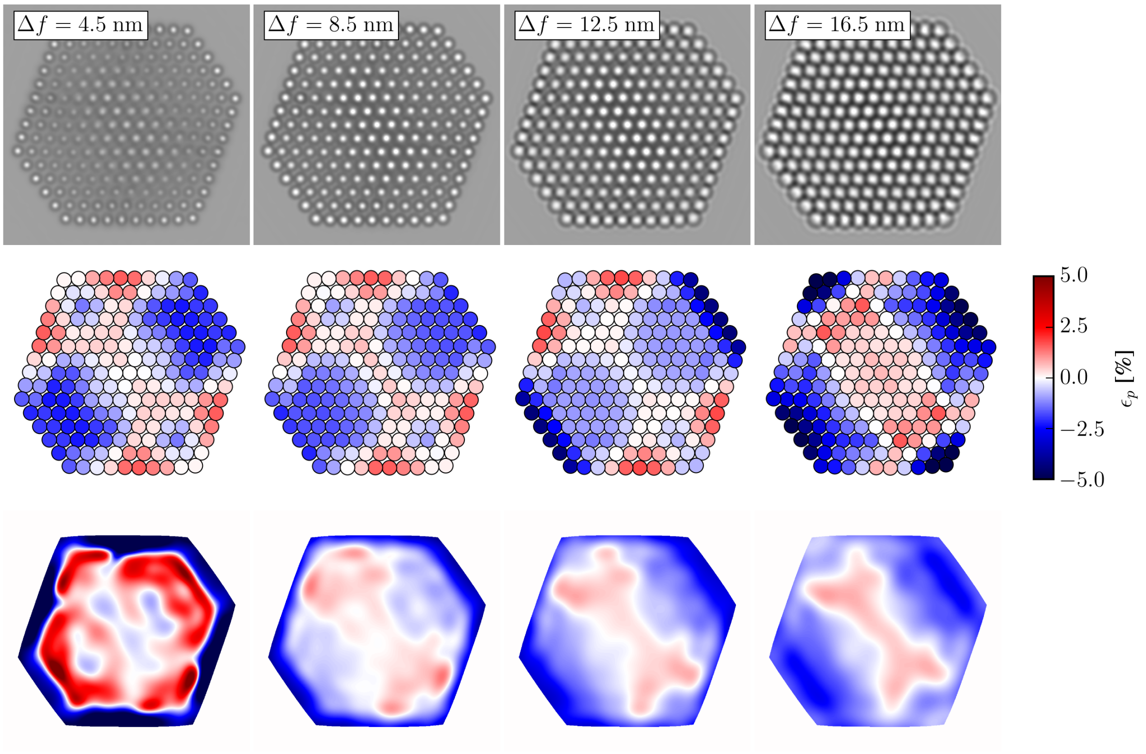

Figure S4: Comparison of the real space analysis described in the main text and analysis using Geometric Phase Analysis (GPA). The largest difference is seen for a defocus $\Delta f = 4.5$ nm, at this defocus the thickness variation across the nanoparticle is visible in the image, and the resulting change in periodicity impacts GPA. At larger defocus the methods agrees reasonably well in the internal part of the particle, however the real space method performs better at the surface.

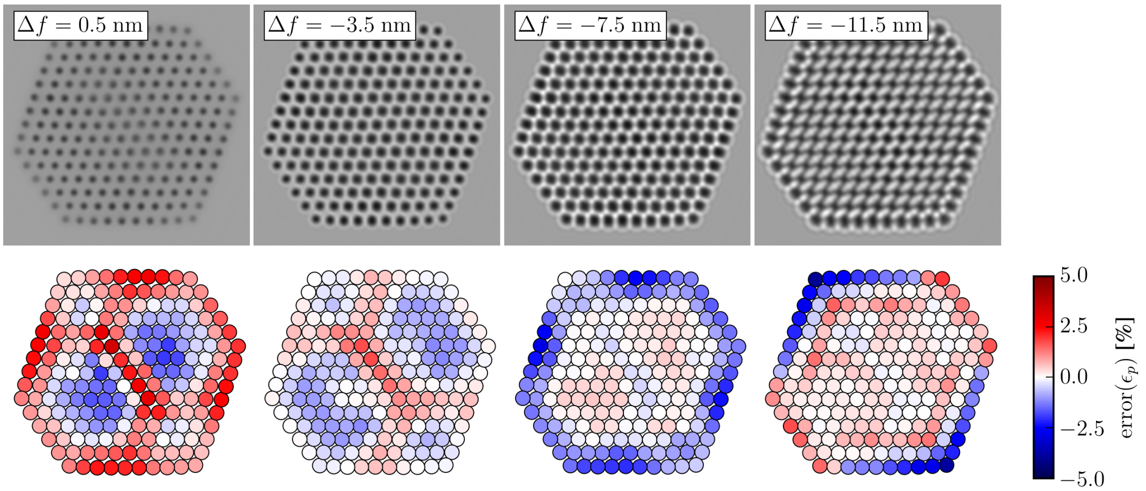

Figure S5: The top row shows simulated images for a nanoparticle with a diameter of 4 nm. The bottom row shows the corresponding distribution of errors in the planar strain. The defocus is different in each column, as indicated in the figure.

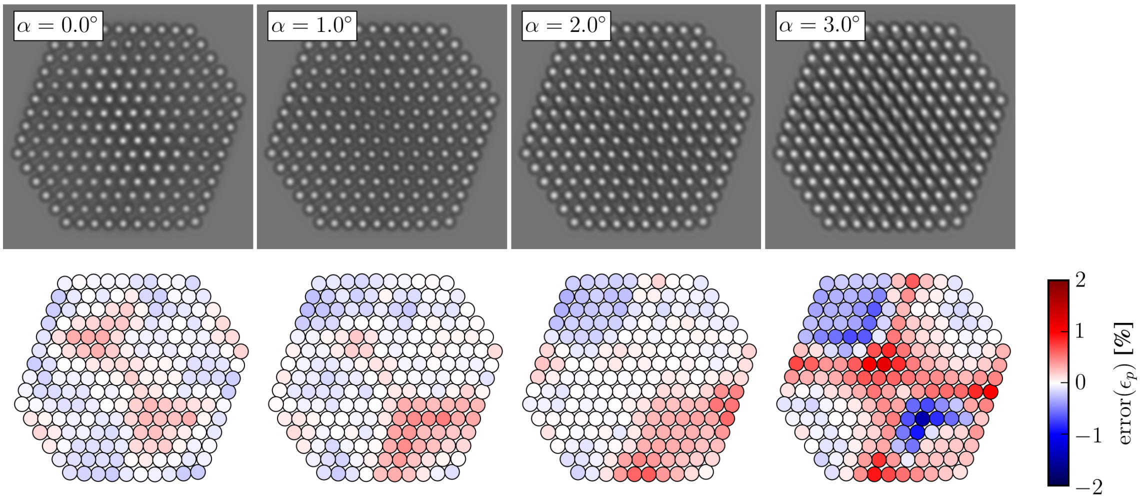

Figure S6: The top row shows simulated images at increasing tilt around the Ω_2-axis for a defocus $\Delta f = 8.5$ nm. The bottom row show the error in the planar strain at each lattice point measured from these images.

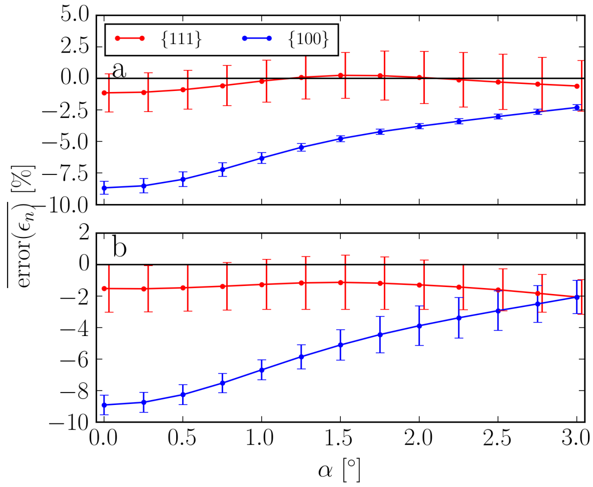

Figure S7: The error in the measured surface relaxations averaged across the facets as a function of tilt, around the axes (a) Ω_1 and (b) Ω_2. The defocus was $\Delta f = 14.5$ nm. The curves are for the {100} and {111} facets, as indicated by the legend.
